# An NS1-F161L Substitution Determines Host-Driven Virulence Enhancement of H5N6 Avian Influenza Virus in Ducks

**DOI:** 10.3390/v18050488

**Published:** 2026-04-23

**Authors:** Yuwei Wu, Zhifan Li, Nuo Xu, Zijun Lu, Yurui Dong, Kunlin Li, Ying Bian, Chenzhi Huo, Tao Qin, Sujuan Chen, Hui Yang, Daxin Peng, Xiufan Liu

**Affiliations:** 1 College of Veterinary Medicine, Yangzhou University, Jiangsu, Yangzhou 225009, China; dx120220179@stu.yzu.edu.cn (Y.W.); mx120230968@stu.yzu.edu.cn (Z.L.); mx120230974@stu.yzu.edu.cn (Z.L.); dx120220192@stu.yzu.edu.cn (Y.D.); 18722988126@163.com (K.L.); by990316@163.com (Y.B.); 13056116999@163.com (C.H.); qintao@yzu.edu.cn (T.Q.); chensj@yzu.edu.cn (S.C.); xfliu@yzu.edu.cn (X.L.); 2 College of Animal Husbandry and Veterinary Medicine, Jiangsu Vocational College of Agriculture and Forestry, Jiangsu, Jurong 212400, China; 3 Jiangsu Co-Innovation Center for the Prevention and Control of Important Animal Infectious Disease and Zoonoses, Jiangsu, Yangzhou 225009, China; 4 Jiangsu Key Laboratory of Zoonosis, Yangzhou University, Jiangsu, Yangzhou 225009, China; 5 Joint International Research Laboratory of Agriculture and Agri-Product Safety, the Ministry of Education of China, Yangzhou University, Jiangsu, Yangzhou 225009, China; 6 Jiangsu Research Centre of Engineering and Technology for Prevention and Control of Poultry Disease, Jiangsu, Yangzhou 225009, China; 7 Jiangsu Interdisciplinary Center for Zoonoses and Biosafety, Yangzhou University, Jiangsu, Yangzhou 225009, China

**Keywords:** H5N6, duck, passage, pathogenicity, mutation

## Abstract

H5 subtype avian influenza virus (AIV) can infect both chickens and ducks, leading to substantial economic losses. Nevertheless, certain strains cause silent infections in ducks. In this study, a goose-origin clade 2.3.4.4h H5N6 AIV was isolated, which caused high mortality in mixed-gender white leghorn chickens but no deaths in mixed-gender mallard ducks. After independent serial in vitro passage in duck embryo fibroblasts (DEFs) and in vivo passage in specific-pathogen-free (SPF) ducks, the DEF-passage 10 (P10) virus induced markedly higher mortality rates and viral loads in SPF ducks compared to the DEF-P1 virus and the original parental virus prior to passage. Similarly, the in vivo-passaged P3 and P4 viruses exhibited significantly higher mortality rates than the P1 virus in SPF ducks, with 100% mortality and markedly increased viral titers in the organs. A whole-genome SNP analysis identified seven high-frequency mutations in the M1, NA and NS1 proteins. The NS1-F161L substitution virus exhibited significantly increased mortality rates, viral loads in multiple tissues, and a robustly induced innate immune response in ducks. Furthermore, dynamic evolutionary variations in the NS1 protein among global H5 avian influenza viruses revealed that the NS1-F161L substitution became dominant in clade 2.3.4.4b viruses in 2021 and subsequent years. Collectively, our findings demonstrate that host-driven adaptation can rapidly increase the pathogenicity of H5N6 AIVs in ducks and identify NS1-F161L as a critical virulence marker. These results offer novel insights relevant to the molecular surveillance, virulence prediction, and risk assessment of circulating H5 AIVs in waterfowl.

## 1. Introduction

H5 subtype avian influenza viruses (AIVs) are important zoonotic pathogens causing severe economic losses in the poultry industry and persistent threats to public health. The viruses encompass numerous neuraminidase (NA) subtypes, including H5N1, H5N2, H5N5, H5N6 and H5N8 AIVs. H5N6 AIVs emerged in 2013 and have spread among wild birds and poultry [1]. Since late 2014, clade 2.3.4.4 H5N6 subtype AIVs have become dominant and spread widely to Japan, China, Europe, and North America. During global dissemination, these viruses continued to evolve and diversify into clade 2.3.4.4a–h [2,3]. Among these, clade 2.3.4.4h was widely prevalent during 2018–2020 and distributed mainly in China, Bangladesh, and Vietnam [4,5]. Concurrently, the frequent reassortment of internal gene segments has contributed to the genetic diversity of H5N6 AIVs, resulting in the emergence of multiple genotypes. The original G1 genotype gave rise to the G1.1 and G1.2 genotypes, with the G1.1 genotype circulating predominantly in ducks and emerging as the dominant lineage [2]. The continuous circulation and evolution of diverse subtypes and genotypes enhance viral cross-species adaptability, pathogenicity and transmission efficiency, thereby further exacerbating the zoonotic risk posed by H5N6 AIVs.

H5 subtype HPAIVs have a broad host range and can infect poultry including chickens and ducks as well as various wild bird species. However, their pathogenicity varies with host species, viral clades and genotypes, and even among different species within the same host group. H5 AIVs typically cause 100% mortality and severe systemic infection in chickens. In contrast, H5N6 viruses exhibit variable pathogenicity in ducks [6,7]. A waterfowl-derived clade 2.3.4.4h H5N6 virus caused no mortality but only mild clinical signs in ducks [8], whereas two clade 2.3.4.4c H5N6 isolates showed markedly different virulence in ducks; the peacock-derived isolate caused high mortality while the duck-derived isolate was non-lethal [9]. Moreover, H5N6 viruses with different genotypes or reassortant gene constellations can display markedly different pathogenicity in ducks [10,11]. Distinct mortality rates are also observed among different H5N6 AIVs in Muscovy ducks and Pekin ducks [12].

Host-driven evolution plays a critical role in AIVs, leading to adaptive mutations across multiple viral gene segments, thereby modulating viral replication, host adaptation, and pathogenicity. Serial passage in host models is a common approach to investigate the host adaptation and pathogenicity of AIVs. A previous study has demonstrated that avirulent AIVs can convert to HPAIVs after serial passage in chickens [13]. Following serial passage of H5 AIVs in Muscovy ducks, the viruses maintained high pathogenicity but acquired stable non-synonymous substitutions in the NS gene, indicating strong host-driven selection [14]. Meanwhile, serial passage of H5N1 and H5N2 AIVs in ducks increased amino acid substitution rates in the HA gene [15]. Further studies have identified multiple molecular markers associated with high pathogenicity in ducks, including a 20-amino-acid deletion in the NA stalk and an 80–84-amino-acid deletion of NS1 [16], as well as several amino acid substitutions determined by comparing viruses with differential virulence such as PA-101G/237E, PA-S224P/N383D, and M1-L43M [17,18,19]. However, the comprehensive linkage between host-driven adaptive mutations and the variable pathogenicity of H5N6 viruses in ducks remains to be fully clarified, which is critical for revealing the adaptive evolution and molecular mechanisms of AIVs in their natural reservoir hosts.

In this study, we serially passaged a clade 2.3.4.4h H5 subtype AIV in vitro and in vivo, evaluated viral pathogenicity before and after passage, and identified adaptive mutations to elucidate the key molecular determinants of viral pathogenicity variation.

## 2. Materials and Methods

### 2.1. Ethics Statements

All experiments involving H5N6 subtype AIVs were conducted in an Animal Biosafety Level 3 (ABSL-3) facility. All animal experiments were approved by the Jiangsu Provincial Experimental Animal Management Committee (Approval No. SYXK-SU-2021-0027) and the Experimental Animal Ethics Committee of Yangzhou University (Approval No.: 202202206 for chicken, Approval date: 28 February 2022; Approval No.:202503190 for ducks, Approval date: 10 March 2025). Laboratory animal housing and experiments were performed strictly in accordance with institutional animal welfare guidelines.

### 2.2. Animals, Cells and Virus

Three-week-old specific-pathogen-free (SPF) mallard ducks were hatched and reared at Yangzhou University from SPF embryonated duck eggs purchased from Harbin Veterinary Research Institute (Harbin, China). Three-week-old SPF white leghorn chickens and SPF embryonated chicken eggs were purchased from Zhejiang Lihua Agricultural Technology Company Limited (Ningbo, China). The SPF ducks weighed approximately 140–190 g and the SPF chickens 170–230 g. All animals were of mixed gender, healthy upon purchase, and not genetically modified. The SPF chickens and ducks were housed separately in negative-pressure, HEPA-filtered isolators under strict biosafety containment. The environmental conditions were maintained at 22–25 °C with 50–60% relative humidity. Isolators for different groups were identical and their positions balanced. SPF embryonated duck eggs were incubated to 11 days of embryonation for the preparation of duck embryo fibroblast (DEF) cells, which were cultured in Dulbecco’s Modified Eagle Medium (DMEM, HyClone, Waltham, MA, USA) containing 1% penicillin-streptomycin and 10% fetal bovine serum (FBS). Human embryonic kidney (HEK) 293T cells and Madin–Darby canine kidney (MDCK) cells were maintained in the same medium formulation. SPF embryonated chicken eggs were incubated to 10 days of embryonation for the preparation of chicken embryo fibroblast (CEF) cells, which were cultured in DMEM containing 1% penicillin-streptomycin and 4% FBS. The H5N6 virus A/Goose/Wuhu/WH0109/2019 was isolated from the oropharyngeal swab of a healthy goose in a live poultry market (LPM), followed by plaque purification in MDCK cells. This isolate served as the parental virus used for all subsequent experiments in this study.

### 2.3. Phylogenetic Analysis

Hemagglutinin (HA) gene sequences of avian-origin (chicken, duck, and goose) H5N6 AIVs from China were downloaded from the GISAID database (https://www.gisaid.org, accessed 12 February 2026). The sequences were aligned using the MAFFT algorithm in PhyloSuite v1.2.2. Phylogenetic analysis was performed using the maximum likelihood (ML) method in IQ-TREE (version 1.6.8), with 10,000 ultrafast bootstrap replicates, 1000 maximum iterations, and minimum correlation coefficient of 0.90 [20]. Phylogenetic trees were visualized using iTOL (version 7) (https://itol.embl.de/, accessed 12 February 2026) for subclade differentiation, and internal gene origins were color-labeled with the iTOL annotation editor.

### 2.4. Virus Titration and Growth Curve

For the HA titer assay, 50 μL of phosphate-buffered saline (PBS) was added to 96-well V-bottom plates (Corning Inc., Corning, NY, USA), and viruses were two-fold serially diluted. Then, 50 μL of 1% chicken red blood cells were added to each well, with incubation at 37 °C for 15 min. HA titer was defined as the highest viral dilution inducing complete hemagglutination.

For the 50% tissue culture infectious dose (TCID_50_) assay, viruses were ten-fold serially diluted and inoculated into 96-well plates seeded with DEF cells, with four replicates per dilution. Plates were incubated at 37 °C in 5% CO_2_ for 72 h. Viral infection was determined by the HA assay, and TCID_50_ values were calculated using the Reed–Muench method [21].

For the 50% egg infectious dose (EID_50_) assay, viruses were ten-fold serially diluted from 10^−6^ to 10^−10^ and inoculated into SPF chicken embryos, with four embryos per dilution. Embryos were incubated at 37 °C. All dead and surviving embryos were tested for HA titer. EID_50_ values were calculated using the Reed–Muench method [21].

For viral growth kinetics, DEF cells were inoculated with each virus at a multiplicity of infection (MOI) of 0.01 and incubated at 37 °C in 5% CO_2_. Cell supernatants were collected at different hours post-infection (hpi), and TCID_50_ values were determined using the Reed–Muench method [21].

### 2.5. Viral Load Quantification

To quantify viral loads, probe-based real-time quantitative polymerase chain reaction (RT-qPCR) was employed. We used Primer Express software (version 3.0.3) to design primers and probes targeting the highly conserved M gene of AIV (Table S1), which were synthesized by Sangon Biotechnology (Shanghai, China). The plasmid containing the M gene was serially diluted to serve as a standard template. Total RNA was extracted using a commercial viral nucleic acid extraction kit (NanoMagBio, Wuhan, China) according to the manufacturer’s instructions. Complementary DNA (cDNA) was synthesized using a one-step reverse transcription kit (TransGen, Beijing, China), followed by amplification via probe-based RT-qPCR with the PerfectStart II Probe qPCR SuperMix (TransGen, Beijing, China). A standard curve was generated by plotting the threshold cycle (Ct) values against base-10 logarithmic concentrations of the serial dilutions.

### 2.6. Serial Passage of WH0109 Virus in DEF Cells and SPF Ducks

In vitro, the WH0109 virus was used to infect DEF cells at an MOI of 0.01. Cell supernatants were harvested at approximately 80% cytopathic effect (CPE), and used to reinfect fresh DEF cells at the same MOI. This process was repeated serially until passage 10 (P10) was obtained.

All animals were housed in the experimental facility for 3 days prior to inoculation to reduce stress from environmental changes. In vivo, five three-week-old SPF ducks were randomly allocated for the P1 group and inoculated intranasally and ocularly with 100 μL of PBS-diluted virus at a dose of 10^6^ EID_50_ via dropwise application using a pipette tip, with gentle handling to avoid physical injury or excessive stress. A total of 30 ducks were used in the SPF-duck-passaged group. Oropharyngeal and cloacal swabs were collected at 3 and 5 days post-infection (dpi), or immediately upon death. Positive swab samples detected by probe-based RT-qPCR were pooled for the next passage, and 100 μL of the pooled sample was inoculated into five new three-week-old SPF ducks intranasally and ocularly. This process was repeated until P5 was obtained.

### 2.7. SNP Sequencing

Lung tissues collected from the ducks infected with DEF-passaged viruses WH0109-P1 and WH0109-P10, as well as lung tissues from SPF ducks at passage levels P1, P3, and P5 during serial in vivo transmission, were subjected to SNP sequencing by Hangzhou LC Bio Technology Co., Ltd. (Hangzhou, China).

Adaptive mutations were identified from the sequencing data using the following rules: (1) Only mutations located in open reading frames were retained. (2) Only non-synonymous amino acid substitutions were included, while synonymous mutations were discarded. (3) Sequencing depth (DP) ≥ 5. Variants with DP < 5 were removed as technical noise.

### 2.8. Construction of High-Frequency Point-Mutated Viruses and Rescue of Recombinant Viruses

Point mutations were introduced into M1, NA, and NS1 plasmids using the Mut Express II Fast Mutagenesis Kit V2 (Vazyme, Nanjing, China). The primers used for mutagenesis are listed in Table S2.

Recombinant viruses were generated as previously described [22]. Briefly, 293T/MDCK co-cultures were co-transfected with rescue plasmids. The remaining gene segments of the rW virus were co-transfected with the point-mutated M1, NA, or NS1 plasmids. The parental virus and point-mutant recombinant viruses were rescued, including rW-M1-T227A, rW-M1-R243W, rW-NS1-A127V, rW-NS1-F161L, rW-NS1-A220V, rW-NA-K251M, and rW-NA-K251I.

### 2.9. Pathogenicity of DEF-Passaged, SPF-Duck-Passaged and Point-Mutant Recombinant Viruses in SPF Ducks

In the pathogenicity assays of the DEF-passaged viruses, the three-week-old SPF ducks were randomly divided into three groups: WH0109-P1 infection group, WH0109-P10 infection group, and PBS control group. Meanwhile in the pathogenicity assays of the point-mutant recombinant viruses, the SPF ducks were randomly divided into eight groups: seven point-mutation recombinant viruses infection groups and PBS control group, both with eight ducks per group. A total of 24 ducks were used in the DEF-passaged group, and 64 ducks in the point-mutation recombinant group. The ducks were inoculated intranasally and ocularly with 100 μL of PBS-diluted virus containing a dose of 10^6^ EID_50_. Three ducks per group were assigned to Group 1 and euthanized at 5 dpi. Tissue samples from the heart, liver, spleen, lung, kidney, and brain were collected. The remaining five ducks per group were assigned to Group 2. Oropharyngeal and cloacal swabs were collected at 1, 3, 5, 7, 10, and 14 dpi. Survival and clinical signs were recorded daily for 14 days.

In the pathogenicity assays of the SPF-duck-passaged viruses, each passage group contained five 3-week-old SPF ducks. Clinical signs and mortality were recorded daily for 5 days. Oropharyngeal and cloacal swabs were collected at 3 and 5 dpi, or immediately upon death. Heart, liver, spleen, lung, kidney, and brain tissues were collected immediately from deceased ducks. Surviving ducks were euthanized at 5 dpi for tissue collection.

For viral quantification in all pathogenicity assays, exactly 0.1 g of each tissue was homogenized at an amplitude of 6.90 m/s for 15 s with 10 s interval for four cycles. Viral loads in all swab and tissue samples were determined by probe-based RT-qPCR and calculated using a standard curve.

The animals were monitored three times daily (every 8 h) for the first 7dpi, and twice daily thereafter until the end of the experiment. After completion of the experiments, all surviving animals were euthanized by manual cervical dislocation in accordance with the AVMA Guidelines for the Euthanasia of Animals (2020 Edition).

### 2.10. Quantification of Immune-Related Genes in Tissue Samples of Ducks Infected with Point-Mutant Recombinant Viruses

Lung tissues from the SPF ducks infected with point-mutant viruses were harvested at 5 dpi for immune-related gene expression analysis. Viral RNA was extracted with TRIzol reagent (Thermo Fisher Scientific, Waltham, MA, USA), and cDNA was synthesized from 1 μg of RNA using a one-step reverse transcription kit (TransGen, China). RT-qPCR was conducted in a 20 μL reaction volume using generated cDNA as template to assess the mRNA levels of *IFN-α*, *IFN-β*, *IL-6*, and *TNF-α*. The mRNA levels of target genes were normalized to the *GAPDH* [23]. All qPCR primer sequences are listed in Table S3 [24,25,26].

### 2.11. Statistical Analysis

All animals were included in the statistical analysis since no unexpected adverse events occurred. Graphs were plotted and statistical analyses were performed using GraphPad Prism V8.3.0 software. Student’s *t*-test was used for comparisons between two groups. Statistical significance was defined as: *p* > 0.05 (ns), *p* < 0.05 (*), and *p* < 0.01 (**).

## 3. Results

### 3.1. Genomic Source Analysis of Internal Genes of Chinese Avian-Origin H5N6 Isolates and Biological Characterization and Phylogenetic of the WH0109 Virus

**Figure 1:**
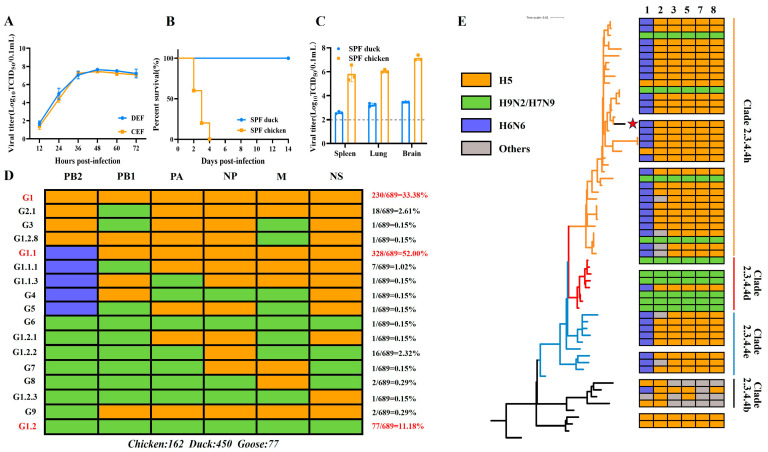
Genotypic source analysis of internal genes of H5N6 AIVs and characterization of the WH0109 virus. (**A**) Growth curves on DEF and CEF cells measured by TCID_50_. (**B**) Pathogenicity of WH0109 virus in SPF chickens and ducks. (**C**) Viral loads in the organs of SPF chickens and ducks. (**D**) Subtypes and genotypes distribution of internal gene segments (PB2, PB1, PA, NP, M, and NS) of H5N6 AIVs from chickens (*n* = 162), ducks (*n* = 450), and geese (*n* = 77) in database. Different colors represent gene subtype origins (H5: orange; H9N2/H7N9: green; and H6N6: blue). Right panel shows genotype proportions, with the top three labeled in red. (**E**) Phylogenetic tree based on the HA gene. Colored blocks denote internal gene sources on the right. Different subclades (Clade 2.3.4.4b, d, e, and h) are color-coded, and the WH0109 virus is marked with a red star. Statistical comparisons were performed by unpaired two-tailed Student’s *t*-test.

We performed plaque purification on the A/Goose/Wuhu/WH0109/2019 (H5N6) strain isolated from an LPM and characterized its basic biological characteristics. The results demonstrated that the WH0109 virus exhibited high HA titers, TCID_50_, and EID_50_ values (Table S4), and replicated efficiently in both CEF and DEF cells (Figure 1A). The virus caused 100% mortality in SPF chickens but no mortality in SPF ducks (Figure 1B). Viral loads in the spleen, lung, and brain at 3 dpi were high in SPF chickens but near the detection limit in SPF ducks (Figure 1C). The pathogenicity assays revealed that WH0109 virus is highly pathogenic in SPF chickens but has a low pathogenicity in SPF ducks.

The complete genome sequences of avian-origin H5N6 isolates (chicken, duck, and goose) in China were retrieved and analyzed from the GISAID database. A total of 687 full-genome isolates were screened, including 162 chicken-origin, 450 duck-origin, and 77 goose-origin viruses. The gene constellations of H5N6 AIVs were classified into 17 distinct genotypes. Genotypes G1, G1.1, G1.1.1, G1.1.3, G1.2, G1.2.1, G1.2.2, G1.2.3, G1.2.8 and G2.1 followed the nomenclature scheme defined by Bi et al. (2016) [2], while genotypes G3-G9 were newly designated. The most prevalent genotype was G1.1, in which the PB2 gene was H6N6-origin, whereas other internal genes were H5-origin. The second common genotype was G1, with all internal genes from H5 viruses, followed by G1.2, with all internal genes from H9 viruses. The remaining genotypes represented a minor fraction (Figure 1D). The phylogenetic analysis of the HA gene and investigation of internal gene origins indicate that WH0109 virus is derived from clade 2.3.4.4h and belongs to the G1.1 genotype (Figure 1E). Thus, we selected the WH0109 virus for further study.

### 3.2. WH0109-P10 Exhibited Enhanced Pathogenicity in SPF Ducks Compared with WH0109-P1

**Figure 2:**
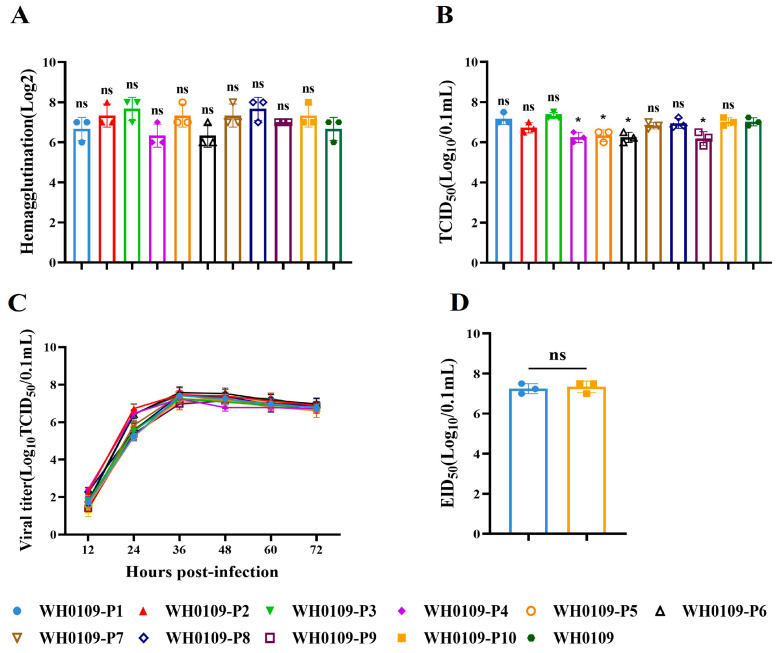
Biological characteristics of WH0109 after serial passage in DEF cells. (**A**–**D**) HA titers (**A**), TCID_50_ (**B**), growth kinetics (**C**) and EID_50_ (**D**) of WH0109 virus after 10 serial passages in vitro. Data are presented as the mean ± SD of three independent experiments. ^ns^ *p* >0.05, * *p* < 0.05.

We determined the HA titer, TCID_50_, growth kinetics and EID_50_ of WH0109 following serial passage in DEF cells. The results showed that only P4, P5, P6 and P9 viruses exhibited significantly reduced TCID_50_ titers compared with the parental WH0109 virus, while no significant differences were observed in the other assays (Figure 2A–D). Notably, all passaged viruses maintained high infectivity titers, indicating that serial passage did not affect the infectivity.

**Figure 3:**
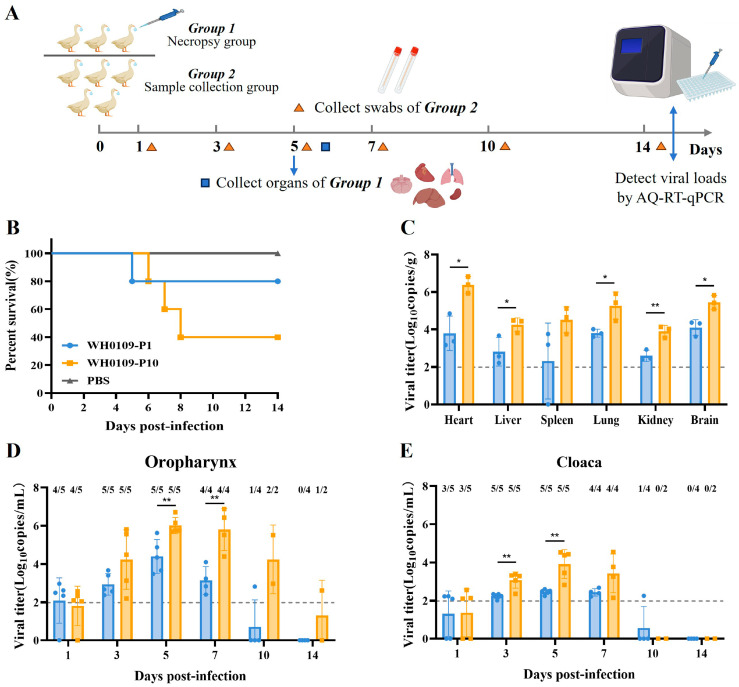
Pathogenicity of in vitro-passaged viruses in SPF ducks. (**A**) Experimental design: Ducks were divided into a necropsy group (*n* = 3) and a sample collection group (*n* = 5). Necropsy group ducks were euthanized at 5 dpi for organ collection. Oropharyngeal and cloacal swabs were collected from the sample collection group at 1, 3, 5, 7, 10 and 14 dpi. Viral loads were detected by probe-based RT-qPCR. (**B**) Survival curves of ducks in the WH0109-P1, WH0109-P10 and PBS control groups. (**C**) Viral loads in various organs of infected SPF ducks. (**D**,**E**) Viral loads in oropharyngeal (**D**) and cloacal (**E**) swabs. Positive/total samples numbers are labeled above each bar. Different colors represent distinct infection groups, and the dashed line indicates the viral load limit of detection. * *p* < 0.05, ** *p* < 0.01.

To determine whether serial passage altered viral pathogenicity in the SPF ducks, the ducks were inoculated with WH0109-P1 or WH0109-P10 at a dose of 10^6^ EID_50_. WH0109-P1 and WH0109-P10 represent the virus after one and ten passages in DEF cells, respectively. Three ducks per group were euthanized at 5 dpi for tissue collection, and the remaining five were monitored for clinical signs and sampled for oropharyngeal and cloacal swabs (Figure 3A). The mortality rate was 60% in the WH0109-P10 group, whereas only 20% mortality was observed in the WH0109-P1 group (Figure 3B). Viral loads of WH0109-P10 were significantly higher than those of WH0109-P1 in all tested tissues except the spleen (Figure 3C). Analysis of swab samples revealed that viral loads of WH0109-P10 were consistently higher than those of WH0109-P1 throughout the observation period. Specifically, viral loads in oropharyngeal swabs differed significantly at 5 and 7 dpi (Figure 3D), whereas cloacal swabs exhibited significant differences at 3 and 5 dpi (Figure 3E).

### 3.3. Continuous Passage in SPF Ducks Significantly Enhanced Pathogenicity and Viral Loads Compared with the First Passage

**Figure 4:**
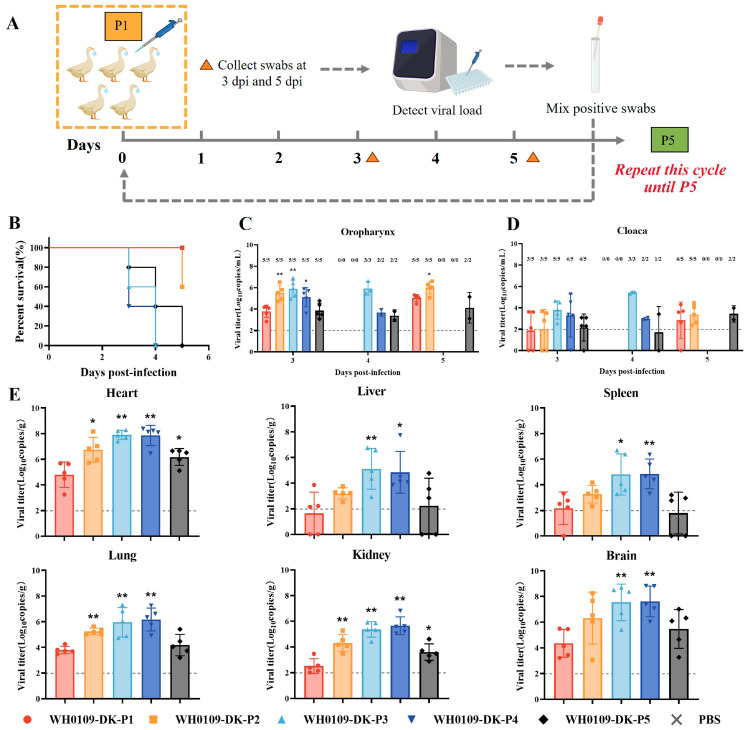
Pathogenicity of viruses serially passaged in SPF ducks. (**A**) Experimental design: SPF ducks (*n* = 5) were inoculated with each passage virus. Oropharyngeal and cloacal swabs were collected at 3 and 5 dpi. Viral loads of all swab samples were detected by probe-based RT-qPCR. Positive swabs were pooled for inoculating the next passage for up to five passages. (**B**) Survival curves of serially passaged viruses in ducks. (**C**,**D**) Viral loads in oropharyngeal (**C**) and cloacal (**D**) swabs from P1 to P5. Positive/total sample numbers are labeled above each bar. (**E**) Viral loads in various organs of SPF ducks from P1 to P5. The dashed line indicates the viral load limit of detection. * *p* < 0.05, ** *p* < 0.01.

To mimic the natural transmission of the virus among ducks in LPMs, the WH0109 virus was serially passaged in the SPF ducks for five generations. The ducks were inoculated at 10^6^ EID_50_, and oropharyngeal and cloacal swabs were collected at 3 and 5 dpi. Positive swabs were pooled for the infection of next passage (Figure 4A). Survival analysis showed that the mortality rate of each passaged group was significantly higher than that of P1. All ducks in the P3, P4, and P5 groups died, with the most rapid mortality observed in the P3 and P4 groups (Figure 4B). Oropharyngeal swab viral loads were significantly increased in P2, P3, and P4 at 3 dpi compared with P1 (Figure 4C,D). Viral loads in all tissues were also significantly higher in all passaged groups than those in P1 (Figure 4E). In summary, serial passage in SPF ducks rapidly increased viral pathogenicity.

### 3.4. Rescue of Point-Mutant Viruses and Evaluation of Their Basic Biological Characteristics

To explore the mechanism underlying pathogenicity differences between in vitro- and in vivo-passaged viruses, SNP sequencing was performed on lung tissues from SPF ducks infected with the in vitro-DEF-cell-passaged viruses WH0109-P1 and WH0109-P10, as well as lung tissues from SPF ducks infected with the in vivo-SPF-duck-passaged viruses WH0109-P1, WH0109-P3, and WH0109-P5. All gene segments were compared to identify different amino acid sites between groups in vitro and in vivo (Table 1). Seven high-frequency mutation sites were identified in both in vitro and in vivo systems: NA-K251M, NA-K251I, M1-T227A, M1-R243W, NS1-A127V, NS1-F161L, and NS1-A220V.

**Figure 5:**
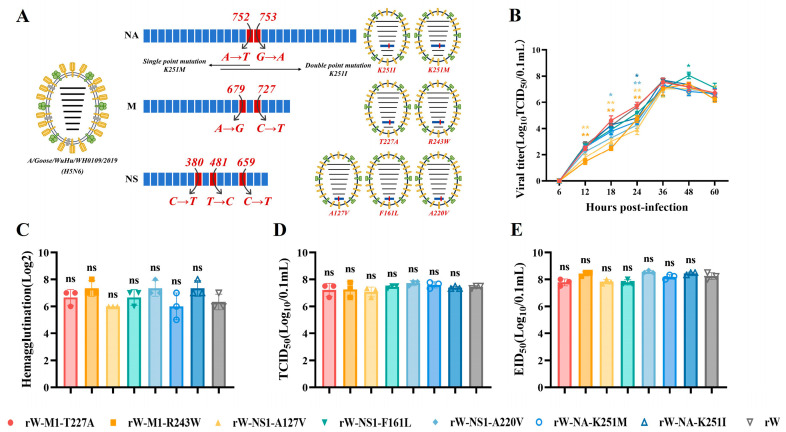
Construction and basic biological characterization of WH0109 point-mutant recombinant viruses. (**A**) Schematic diagram of point-mutant virus construction. Mutation positions, nucleotide changes, and single/double mutations are labeled on NA, M and NS gene segments. The corresponding recombinant viruses and amino acid mutation sites are shown on the right. (**B**–**E**) growth kinetics (**B**), HA titer (**C**), TCID_50_ (**D**), and EID_50_ (**E**) of point-mutant recombinant viruses. Different colors represent distinct infection groups. Data are presented as the mean ± SD of three independent experiments. Statistical comparisons were performed using unpaired *t*-test. ^ns^ *p* >0.05, * *p* < 0.05, ** *p* < 0.01.

Subsequently, point mutations were separately introduced into the NA, M1, and NS1 genes, and recombinant viruses were rescued using the AIV reverse genetics system (Figure 5A). Growth kinetics in DEF cells showed that, compared with the parental rW virus, the viral replication of rW-M1-R234W and rW-NS1-A127V was significantly reduced at 12–24 hpi; rW-NS1-A220V was reduced at 18–24 hpi and rW-NA-K251I was reduced at 24 hpi; whereas rW-NS1-F161L displayed significantly higher replication at 48 h (Figure 5B). Measurements of the HA titers, TCID_50_, and EID_50_ assays revealed no significant differences between point-mutant viruses and rW virus, although all viruses maintained high replication titers (Figure 5C–E). Collectively, despite the discrepant replication kinetics at early infection stages, all point-mutant viruses retained efficient replication capacity in vitro.

### 3.5. rW-NS1-F161L Exhibited Enhanced Pathogenicity in SPF Ducks

**Figure 6:**
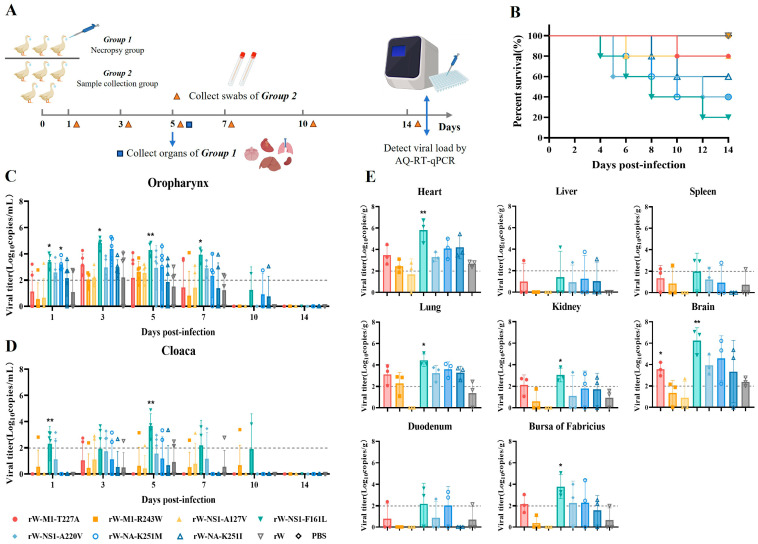
Pathogenicity of point-mutant recombinant viruses in SPF ducks. (**A**) Experimental design: Ducks were divided into a necropsy group (*n* = 3) and a sample collection group (*n* = 5). Necropsy group ducks were euthanized at 5 dpi for organ collection. Oropharyngeal and cloacal swabs were collected from the sample collection group at 1, 3, 5, 7, 10 and 14 dpi. Viral loads were detected by probe-based RT-qPCR. (**B**) Survival curves of point-mutant recombinant virus and PBS control groups. (**C**,**D**) Viral loads in oropharyngeal (**C**) and cloacal (**D**) swabs. (**E**) Viral loads in various organs of infected SPF ducks. Different colors represent distinct infection groups. * *p* < 0.05, ** *p* < 0.01.

To investigate whether point mutations increased viral pathogenicity in the SPF ducks, the ducks were inoculated with seven point-mutant viruses and the rW virus at a dose of 10^6^ EID_50_. Three ducks per group were euthanized at 5 dpi for tissue collection, and the remaining five were sampled for oropharyngeal and cloacal swabs (Figure 6A). The results showed that the rW-NS1-F161L group exhibited the strongest pathogenicity, with a mortality rate of 80%, followed by the rW-NA-K251M and rW-NS1-A220V groups, each with 60% mortality, whereas rW showed 100% survival in SPF ducks (Figure 6B). Viral loads in the oropharyngeal swabs of the rW-NS1-F161L group were significantly higher at 1, 3, 5, and 7 dpi than in the rW group, with cloacal swabs elevated at 1 and 5 dpi. rW-NA-K251M and rW-NS1-A220V also exhibited relatively high viral loads (Figure 6C,D). Consistent with the swab results, the rW-NS1-F161L group displayed the highest viral loads, with significantly higher levels in the heart, lung, kidney, brain, and bursa of fabricius compared with rW group (Figure 6E).

**Figure 7:**
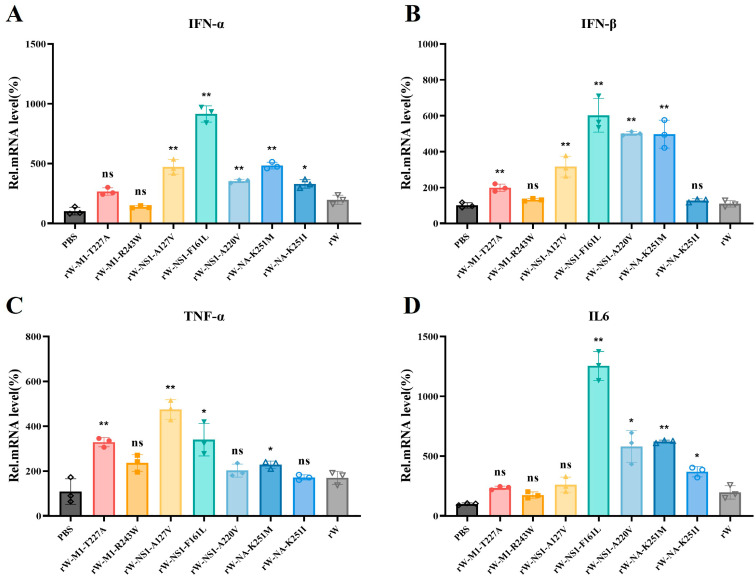
Cytokine gene expression of SPF ducks infected with point-mutant recombinant viruses. (**A**–**D**) qPCR analysis of *IFN-α* (**A**), *IFN-β* (**B**), *TNF-α* (**C**) and *IL-6* (**D**) (*n* = 3 per group). Data were normalized to *GAPDH*, presented as the mean ± SD, and relative to the PBS control (set as 100%). Different colors represent distinct infection groups. Statistical comparisons were performed using unpaired *t*-test. ^ns^ *p* >0.05, * *p* < 0.05, ** *p* < 0.01.

We further evaluated the mRNA levels of various cytokines in lung tissues from the infected and control groups using RT-qPCR. Compared with the control group, the rW-NS1-F161L group showed approximately a 10-fold increase in *IFN-α* mRNA and a 6-fold increase in *IFN-β* mRNA (Figure 7A,B). In addition, the pro-inflammatory cytokines *TNF-α* and *IL-6* were also significantly upregulated, with *IL-6* exhibiting a 12-fold increase (Figure 7C,D). These results indicated that infection with the rW-NS1-F161L recombinant virus triggers a stronger innate antiviral response and elevated inflammatory cytokine production in SPF ducks.

Meanwhile, we investigated the regulation of innate immunity in DEF cells infected with the rW-NS1-F161L recombinant virus at 6, 12, 24, and 48 hpi. Compared with the rW virus, the NS1-F161L mutant induced significantly lower expression of *IFN-α* and *IFN-β* during the early stage of infection. The induction of *IFN-α* was significantly reduced at 6 h and 24 hpi, while *IFN-β* expression was markedly suppressed at 6 h and 12 hpi (Figure S2A,B). In contrast, when compared with the rW virus, the NS1-F161L mutant induced significantly higher levels of *TNF-α* at 12 h and 48 hpi, as well as *IL-6* at 6 and 12 hpi (Figure S2C,D). Collectively, these findings demonstrate that the NS1-F161L mutation significantly suppresses the induction of type I interferons but promotes the expression of pro-inflammatory cytokines in DEF cells during the early phase of infection.

### 3.6. GISAID Database Analysis of Amino Acid Sites During Serial Passage

**Figure 8:**
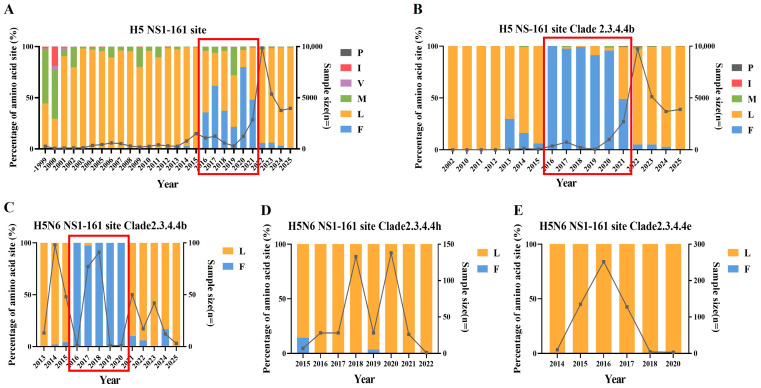
GISAID database analysis of NS1-161 site (**A**,**B**) Amino acid frequency distributions at NS1-161 site in all global avian H5 AIVs (**A**) and in the clade 2.3.4.4b of H5 AIVs (**B**). (**C**–**E**) Amino acid frequency distributions at NS1-161 site in global avian H5N6 AIVs of clade 2.3.4.4b (**C**), clade 2.3.4.4h (**D**) and clade 2.3.4.4e (**E**). Red boxes indicate the time period during which amino acid changes occurred.

We analyzed the NS1-161 site of global H5 avian AIVs in the GISAID database. Across all H5 subtype AIVs, 161L predominated prior to 2016. Between 2016 and 2021, the frequency of 161F increased markedly and was mainly associated with clade 2.3.4.4b. Since 2021, 161L has re-emerged as the dominant residue (Figure 8A,B). Within H5N6 AIVs, the same trend is evident in clade 2.3.4.4b (Figure 8C). In clade 2.3.4.4h, 161F was only sporadically detected in 2015 and 2019, while 161L predominated in other years (Figure 8D). Similarly, clade 2.3.4.4e was also dominated by 161L (Figure 8E).

## 4. Discussion

Host-driven adaptive evolution is a key driver of AIV virulence and host range expansion, with waterfowl serving as a pivotal niche for viral diversification. In this study, we systematically investigated the adaptive evolution of an H5N6 AIV that is highly pathogenic in chickens but of low pathogenicity in ducks using parallel in vitro and in vivo passage in ducks, and found that the virus exhibited significantly increased pathogenicity following serial passage. We identified the NS1-F161L substitution as a critical molecular determinant of enhanced virulence, and this observation advances our understanding of H5N6 adaptation to its natural duck host.

Serial passage of AIVs within a host typically drives alterations in virulence and host adaptation. In diverse viral subtypes and host models, serial passage experiments have revealed the emergence of high-frequency mutations and enhanced replicative capacity [27,28]. The mouse model has been a commonly used animal model to study the host adaptation and pathogenicity of influenza A viruses in general. Previous studies demonstrated that serial lung passage of human seasonal H3N2 influenza viruses induced 14 amino acid mutations across multiple genes, elevating polymerase activity and pathogenicity in mice [29]. Similar approaches have also been employed for AIVs in avian hosts. In chicken hosts, non-pathogenic viruses acquire multi-basic cleavage sites in the HA gene through serial passage, resulting in their conversion to highly pathogenic viruses with lethal outcomes [13]. In duck hosts, serial in vivo passage of the chicken-origin H5N1 and H5N2 virus led to the accumulation of high-frequency mutations in the HA gene, accompanied by enhanced tissue tropism [15]. In this study, the biological characteristics of the virus remained unchanged after serial passage in DEF cells, whereas distinct phenotypic differences in pathogenicity were observed following challenge in SPF ducks. This discrepancy between in vitro and in vivo outcomes may be attributed to the absence of a complete innate immune system and structured tissue microenvironment in in vitro systems, which are critical determinants of viral pathogenicity in vivo [30]. In addition, adaptive mutations were detected in the PB2, HA, NP, NA, M1, M2, and NS1 genes during the sequencing analysis of lung tissues from SPF ducks infected after serial in vitro passage and from ducks undergoing serial in vivo passage. The high-frequency mutations in both in vitro and in vivo passage were concentrated in NA, M1, and NS1. These mutations markedly enhanced viral replication and tissue invasiveness, thereby facilitating the transition from low to high pathogenicity. Waterfowl exhibit a relatively subdued antiviral inflammatory response, providing a permissive environment for sustained viral replication and evolution. This characteristic renders waterfowl critical intermediaries in viral host adaptation and pathogenicity evolution [31,32].

During the long-term evolution of AIVs in waterfowl, adaptive amino acid mutations in viral gene segments can enhance both replication efficiency and tissue tropism. Several studies have reported that the S224P and N383D mutations in PA contribute to the highly virulent phenotype of H5N1 AIVs in ducks [18]. Using reverse genetics, recombinant viruses were constructed to demonstrate that M1 L43M is a key determinant underlying increased pathogenicity of H5N1 AIVs in ducks [19]. In addition, the PA mutations 101G and 237E in H5N1 HPAIVs enhance viral replication, polymerase activity, and pathogenicity in ducks [17]. In this study, SNP sequencing identified seven high-frequency substitutions: M1-T227A, M1-R243W, NA-K251M, NA-K251I, NS1-A127V, NS1-F161L, and NS1-A220V. Among these, NS1-F161L was identified as the key adaptive mutation responsible for markedly increased pathogenicity in ducks. Furthermore, this mutation facilitated systemic infection in ducks, with efficient replication in various tissues, particularly high viral loads in the heart, lung, and brain. Consistent with previous studies, H5 AIV infection in ducks exhibited multi-organ tropism, with elevated viral titers in the lung, kidney, brain, pancreas, accompanied by marked neurological signs [33,34]. The 100% mortality observed in ducks is likely mediated by the synergistic effects of multiple adaptive mutations. We also identified several other substitutions, including NA-K251M/I, M1-T227A/R243W, and NS1-A127V/A220V, that enhance viral pathogenicity. NS1-F161L acts as the predominant virulence determinant by promoting viral replication and pathogenicity and inducing excessive innate immune activation, while the other mutations may act cooperatively to increase virulence. Thus, the adaptive changes may help the virus overcome host restrictions and establish efficient systemic infection and transmission.

We evaluated the innate immune responses of ducks infected with point-mutant recombinant viruses. The NS1-F161L mutation induced a stronger innate immune response, with significantly higher pro-inflammatory cytokines including IFN-α, IFN-β, IL-6 and TNF-α than the parental virus. A major function of influenza virus NS1 is to antagonize the production and signaling of type I interferons, thereby facilitating efficient viral replication [35,36]. However, the highly pathogenic mutant virus induced significantly elevated interferon levels at 5 dpi in this study. This apparent contradiction may be explained by the enhanced replication and systemic tissue dissemination of the mutant H5N6 virus, which triggers increased vascular permeability, multi-organ damage, and ultimately excessive release of type I interferons and pro-inflammatory cytokines [37,38]. These mechanisms likely account for the high-level cytokine expression observed in H5N6-infected ducks, which is driven by enhanced viral replication in major tissues and organs. Notably, the recombinant NS1-F161L virus also showed enhanced replication in DEF cells at 48 h, and suppressed interferon expression while promoting pro-inflammatory cytokine production at the early cellular stage of infection. It appears that this substitution improves not only immune evasion in vivo, but also baseline replication fitness at the cellular level. Given that NS1 is a well-known interferon antagonist, the F161L mutation may exert critical immunomodulatory effects by interfering with RIG-I or NF-κB signaling pathways to suppress interferon production. These pathways are well-documented targets of influenza A virus NS1 proteins, which block TRIM25-mediated RIG-I ubiquitination and inhibit p65 nuclear translocation to counteract host innate immunity [39,40].

A previous structure-guided functional study demonstrated that residue 161 of the NS1 protein in human influenza A viruses is located within the p85β-binding interface and undergoes dynamic evolution during viral circulation in humans, and variations at this position modulate viral virulence by altering NS1-p85β interaction [41]. Phylogenetic expansion, evolutionary divergence, and genetic reassortment of AIVs directly drive dynamic shifts in the frequency of key adaptive mutations [42,43]. GISAID database analysis revealed a pronounced temporal and lineage-dependent pattern at NS1-161 residue among global H5 AIVs. Notably, the intercontinental spread of clade 2.3.4.4b viruses via migratory birds began in 2016 [44], which coincides with our database observation that 161F increased markedly during 2016–2021, predominantly within clade 2.3.4.4b. In January 2020, a new epidemic wave of clade 2.3.4.4b emerged, subsequently diverging into two branches and causing widespread outbreaks in poultry and wild birds globally [45,46,47]. Consistently, our analysis identified a trend reversal, with 161L re-establishing predominance after 2021. This temporal concordance suggests that adaptive mutations in H5 AIVs may facilitate the evolution of clade 2.3.4.4b. This pattern is likely driven by host adaptation, environmental conditions and immune escape [48,49,50]. The dynamic fluctuation of NS1-161 in clade 2.3.4.4b contrasts with the stable predominance of 161L in clades 2.3.4.4h and 2.3.4.4e, highlighting the clade-specific adaptive evolution of H5 AIVs and emphasizing the necessity for continuous surveillance and epidemic preparedness [51]. To determine whether other mutation sites exhibit dynamic changes, we performed database analysis and found that the M1-227 site was dominated by 227A (Figure S1A), while the M1-R243W and NS1-A127V mutation patterns observed in our study were rarely detected in the database (Figure S1B,D). Despite the NA1-K251M/I and NS1-A220V mutations not being the predominant variants in the database, they still exerted effects on viral pathogenicity (Figure S1C,E).

In conclusion, our study defines a molecular mechanism by which host-driven adaptation enhances H5N6 HPAIV virulence in ducks, identifying NS1-F161L as a critical virulence determinant. These findings advance our understanding of AIV adaptive evolution in natural reservoirs and provide essential insights for the development of improved molecular surveillance tools, virulence prediction models, and control strategies to mitigate the threat of H5N6 HPAIVs to animal and public health.

## Figures and Tables

**Table 1:** Differential amino acid sites in lung tissues between in vitro P1 and P10 and in vivo P1, P3 and P5.

Gene	PB2	PB1	PA	HA	NP	NA	M1	M2	NS1	NS2
Amino acid mutation in vitro	/	/	/	H486Y	/	K251M ^a^ K251I ^a^	T227A ^a^ R243W ^a^	R45C H90Y	T86I A127V ^a^ F161L ^a^ A172V A220V^a^	/
Amino acid mutation in vivo	G347C T351I V356A A370V T378I I648L	/	/	/	V194I ^b^ D375E N473H ^c^	Q216H K251M ^a^ K251I ^a^ D375N	P54L R72W S195L T227A ^a^ R243W ^a^	P25S	T56I T76A A127V ^a^ F129L ^c^ F161L ^a^ R215W ^c^ A220V ^a^	/

a: Nucleotide and corresponding amino acid mutation sites shared by both in vivo and in vitro passages; b: Nucleotide and corresponding amino acid mutation sites unique to P3; c: Nucleotide and corresponding amino acid mutation sites shared by P3 and P5.

## Data Availability

The genomic sequences of WH0109 virus are available in GenBank under the accession numbers (PZ097288 for PB2, PZ097289 for PB1, PZ097290 for PA, PZ097291 for HA, PZ097292 for NP, PZ097293 for NA, PZ097294 for M, and PZ097295 for NS).

## Supplementary Materials

The following supporting information can be downloaded at https://www.mdpi.com/article/10.3390/v18050488/s1, Figure S1: GISAID database analysis of SNP mutation sites; Figure S2: Cytokine gene expression of DEF cells infected with rW-NS1-F161L recombinant viruses; Table S1: Primers and probes sequences used in the probe-based RT-qPCR; Table S2: Primers for construction of the recombination plasmids; Table S3: Primer sequences used in RT-qPCR; Table S4: Biological characteristics of WH0109 virus; Table S5: Differential nucleotide and amino acid sites in the 8 genomic segments of DEF-passaged WH0109-P1 and WH0109-P10 viruses from infected duck lung tissues; Table S6: Differential nucleotide and amino acid sites in the 8 genomic segments of SPF duck-passaged WH0109-P1 and WH0109-P3 viruses; Table S7: Differential nucleotide and amino acid sites in the 8 genomic segments of SPF duck-passaged WH0109-P1 and WH0109-P5 viruses.
