# Supplementary material for: An NS1-F161L Substitution Determines Host-Driven Virulence Enhancement of H5N6 Avian Influenza Virus in Ducks
Source: Viruses. 2026 Apr 23;18(5):488. doi: 10.3390/v18050488 (PMC13211696; doi:10.3390/v18050488)
Supplement: Supplementary file 1 [file viruses-18-00488-s001.zip › viruses-4268800-supplementary.pdf]

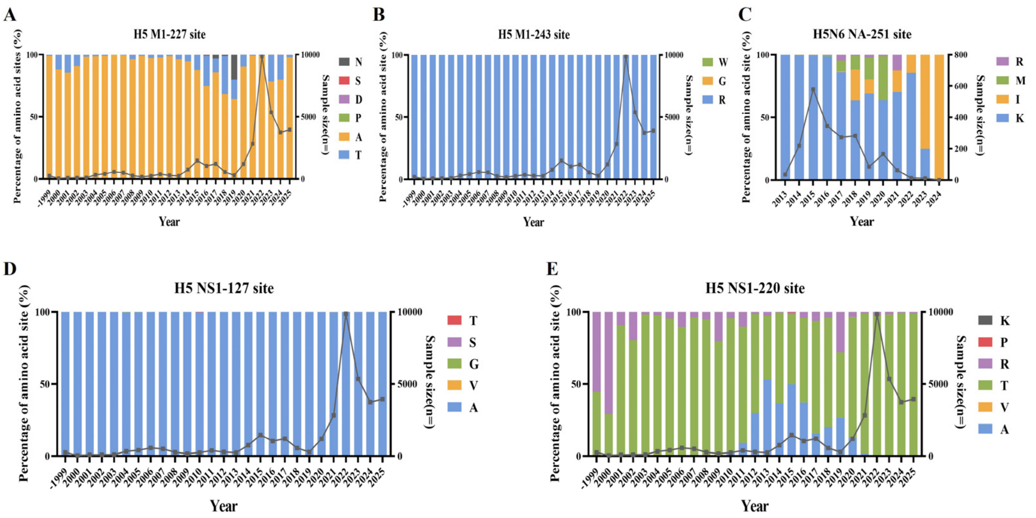

Figure S1. GISAID database analysis of SNP mutation sites

(A and B) Amino acid frequency distributions at M1-227 **(A)** and M1-243 **(B)** sites in global avian H5 AIVs. **(C)** Amino acid frequency distributions at NA-251 site in global avian H5N6 AIVs. **(D and E)** Amino acid frequency distributions at NS1-127 **(D)** and NS1-220 **(E)** sites in global avian H5 AIVs.

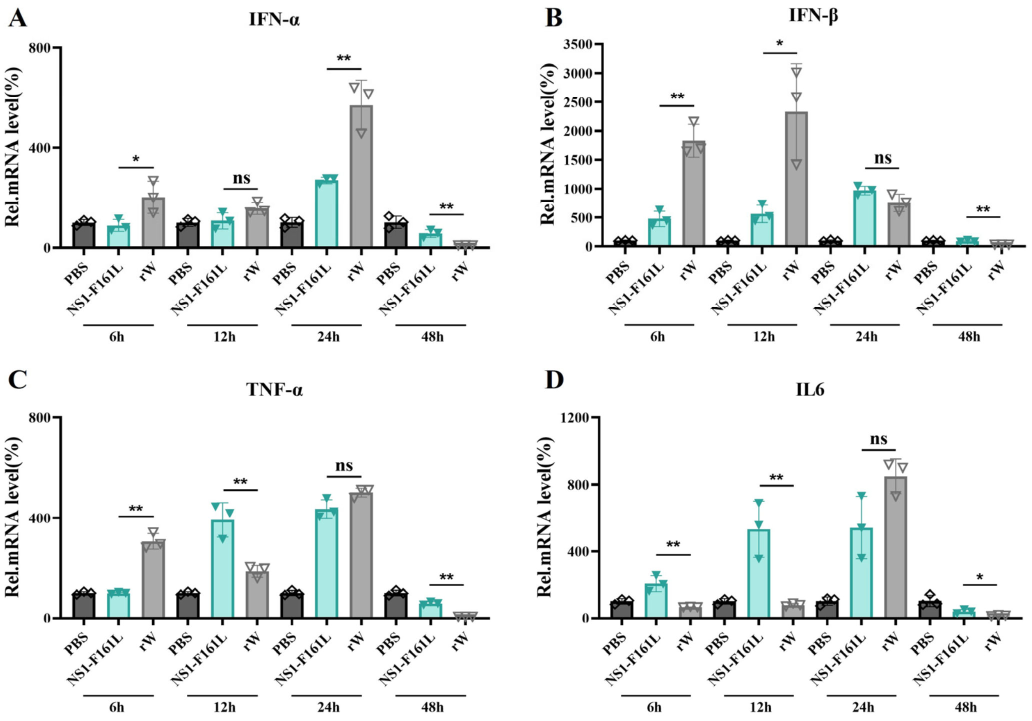

Figure S2. Cytokine gene expression of DEF cells infected with rW-NS1-F161L recombinant viruses

(A-D) qPCR analysis of *IFN-α* **(A)**, *IFN-β* **(B)**, *TNF-α* **(C)** and *IL-6* **(D)** in DEF cells. Data were normalized to *GAPDH*, presented as the mean ± SD, and relative to the PBS control (set as 100%). Statistical comparisons were performed using unpaired *t*-test. $^{ns}P > 0.05$、$^{*}P < 0.05$, $^{**}P < 0.01$

Table S1. Primers and probes sequences used in the probe-based RT-qPCR

Primers and probes	Sequence 5'-3'
Forward Primers	CTTTAKCCAYTCCATGAGAGC
Reverse Primers	CTTCTAACCGAGGTCGAAACG
Probe	FAM-CCTCAAAGCCGAGATC-MGB

Table S2. Primers for construction of the recombination plasmids

	**Forward primer 5'-3'**	**Reverse primer 5'-3'**
rW-M1-T227A	ATTCTAGTGCTGGTCTGAGA GACAATCTTCTTGAA	CAGACCAGCACTAGAATTAG GATGAGTCCCAATTGT
rW-M1-R243W	ACCAGAAATGGATGGGAGTG CAGATGCAGCGA	TCCCATCCATTTCTGGTAGGC CTGCAAATTTT
rW-NS1-A127V	GCATCATACTGAAAGTAAATT TCAGTGTGATTTTTGGCC	TACTTTCAGTATGATGCTTTT ATCCATTATTG
rW-NS1-F161L	TACCTTCTCTTCCAGGACATA CTGGCGAGGAT	TCCTGGAAGAGAAGGTAATG GTGAGATTTCTCCC
rW-NS1-A220V	GGCGAGAGTAATTGAGTCAG AAGTTTGAAGAAATAAGG	ACTCAATTACTCTCGCCAGTT TCCGTTTCTGA
rW-NA-K251M	CAAAGAGGGAATGATACAAA AAACTGAAGAACTGCAAGG	GTATCATTCCCTCTTTGAAGT AGATTATCTTAGTAGC
rW-NA-K251I	CAAAGAGGGAATAATACAAA AAACTGAAGAACTGCAAGG	TGTATTATTCCCTCTTTGAAG TAGATTATCTTAGTAGC

Table S3. Primer sequences used in RT-qPCR

Gene	Sequence 5’-3’	NCBI accession number or Reference
IFN-α	F: CAACGACACGCAGCAAGC<br>R: GGGTGTCGAAGAGGTGTTGG	[1]
IFN-β	F: ACGCTAATACGGCGGATGAA<br>R: GGGCAGTCATAAGGTGCAGA	NM_001310827.2
IL6	F: GTGCGAGAAGTTCACCGTCTG<br>R: TCGTCGAAGCCAGCCAGGAG	XM_027450925.3
TNF-α	F: TCAGCTGGCTAAGACCGTGG<br>R: TTGCAGTTAGGTGACGCTGA	[2]
GAPDH	F: CCACTTCCGGGGCACTGTCA<br>R: AGCACCAGCATCTGCCCACT	[3]

References

1. Figueroa, T.; Bessière, P.; Coggon, A.; Bouwman, K.M.; van der Woude, R.; Delverdier, M.; Verheije, M.H.; de Vries, R.P.; Volmer, R. The Microbiota Contributes to the Control of Highly Pathogenic H5N9 Influenza Virus Replication in Ducks. *Journal of virology* **2020**, *94*, e00289-00220, doi:10.1128/jvi.00289-20.
2. Yang, H.; Wang, Y.; Jin, S.; Pang, Q.; Shan, A.; Feng, X. Dietary resveratrol alleviated lipopolysaccharide-induced ileitis through Nrf2 and NF-κB signalling pathways in ducks (Anas platyrhynchos). *Journal of animal physiology and animal nutrition* **2022**, *106*, 1306-1320, doi:10.1111/jpn.13657.
3. Bessière, P.; Figueroa, T.; Coggon, A.; Foret-Lucas, C.; Houffschmitt, A.; Fusade-Boyer, M.; Dupré, G.; Guérin, J.L.; Delverdier, M.; Volmer, R. Opposite Outcomes of the Within-Host Competition between High- and Low-Pathogenic H5N8 Avian Influenza Viruses in Chickens Compared to Ducks. *Journal of virology* **2022**, *96*, e0136621, doi:10.1128/jvi.01366-21.

Table S4. Biological characteristics of WH0109 virus

**Virus**	**HA titer (Log_2)**	**$TCID_{50}$ (Log_{10}/0.1mL)**	**EID_{50} (Log_{10}/0.1mL)**
A/Goose/Wuhu/WH0109/2019(H5N6)	6.33±0.47	6.89±0.15	7.35±0.11

Table S5. Differential nucleotide and amino acid sites in the 8 genomic segments of DEF-passaged WH0109-P1 and WH0109-P10 viruses from infected duck lung tissues

Gene	PB2	PB1	PA	HA	NP	NA	M1	M2	NS1	NS2
Nucleotide mutation	/	/	/	<u>C1456T</u>	G1281A	T687G<br>T702C<br>T723A<br><u>A752T</u><br><u>G753A</u><br>G846A	A216G<br>T327C<br>G522A<br>T669C<br><u>A679G</u><br>A690G<br><u>C727T</u>	C96T<br><u>C133T</u><br><u>C268T</u>	C336T<br>C369T<br>C555T<br>C649T<br><u>C257T</u><br><u>C380T</u><br><u>T481C</u><br><u>C515T</u><br><u>C659T</u>	G246A
Amino acid mutation	/	/	/	H486Y	/	K251M<br>K251I	T227A<br>R243W	R45C<br>H90Y	T86I<br>A127V<br>F161L<br>A172V<br>A220V	/

__： Nucleotide mutations leading to nonsynonymous amino acid substitutions

Table S6. Differential nucleotide and amino acid sites in the 8 genomic segments of SPF duck-passaged WH0109-P1 and WH0109-P3 viruses

Gene	PB2	PB1	PA	HA	NP	NA	M1	M2	NS1	NS2
Nucleotide mutation	T921C<br>T949C	/	/	/	C406T<br>A486G<br>G519A<br>G580A<br>A597G<br>A1417C	T723A<br>A752T<br>G753A<br>G846A	C435T<br>T669C<br>A679G<br>A690G	/	G105A<br>C141T<br>A183G<br>C336T<br>C414T<br>C380T<br>T385C<br>T481C<br>C643T	G246A
Amino acid mutation	/	/	/	/	V194I<br>N473H	K251M<br>K251I	T227A	/	A127V<br>F129L<br>F161L<br>R215W	/

__: Nucleotide mutations leading to nonsynonymous amino acid substitutions

Table S7. Differential nucleotide and amino acid sites in the 8 genomic segments of SPF duck-passaged WH0109-P1 and WH0109-P5 viruses

Gene	PB2	PB1	PA	HA	NP	NA	M1	M2	NS1	NS2
Nucleotide mutation	T42C<br>G114A<br>T435G<br>G885A<br>G897A<br>T921C<br>T949C<br>A1065G<br>A1113C<br>A1920G<br>G2193A<br><u>G1039T</u><br><u>C1052T</u><br><u>T1067C</u><br><u>C1109T</u><br><u>C1133T</u><br><u>A1942C</u>	G204A<br>T1143C<br>T1389G<br>C1428T<br>A1437G<br>A1794G	/	C66T<br>C1182T<br>A1272G<br>G1287A<br>C1317T<br>T1560G<br>C1585T<br>C1602T<br>C1635T	A72G<br>C141T<br>C235T<br>C282T<br>C393T<br>A486G<br>A597G<br>G738A<br>T774C<br>G777A<br>T1089C<br><u>C1125A</u><br>G1281A<br><u>A1417C</u>	A642G<br><u>A648C</u><br>T687G<br>T702C<br>T723A<br><u>A752T</u><br><u>G753A</u><br>G846A<br>G870A<br>T1059A<br><u>G1123A</u>	G69A<br>C72T<br>T327C<br>A333G<br>C435T<br>G459A<br>G489A<br>G522A<br>T669C<br>A690G<br><u>C161T</u><br><u>C214T</u><br><u>A216G</u><br><u>C584T</u><br><u>A679G</u><br><u>C727T</u>	<u>C73T</u><br>C96T	G105A<br>C141T<br>A183G<br>T207C<br>C336T<br>C555T<br><u>C167T</u><br><u>A226G</u><br><u>C380T</u><br><u>T385C</u><br><u>T481C</u><br><u>C637T</u><br><u>C659T</u>	G246A
Amino acid mutation	G347C<br>T351I<br>V356A<br>A370V<br>T378I<br>I648L	/	/	/	D375E<br>N473H	Q216H<br>K251M<br>K251I<br>D375N	P54L<br>R72W<br>S195L<br>T227A<br>R243W	P25S	T56I<br>T76A<br>A127V<br>F129L<br>F161L<br>R215W<br>A220V	/

__: Nucleotide mutations leading to nonsynonymous amino acid substitutions
